# Metabolic Effects of Acute Thiamine Depletion Are Reversed by Rapamycin in Breast and Leukemia Cells

**DOI:** 10.1371/journal.pone.0085702

**Published:** 2014-01-15

**Authors:** Shuqian Liu, Sumitra Miriyala, Mignon A. Keaton, Craig T. Jordan, Christina Wiedl, Daret K. St. Clair, Jeffrey A. Moscow

**Affiliations:** 1 Department of Pediatrics, University of Kentucky College of Medicine, Lexington, Kentucky, United States of America; 2 Graduate Center for Toxicology, University of Kentucky College of Medicine, Lexington, Kentucky, United States of America; 3 Metabolon, Inc, Durham, North Carolina, United States of America; 4 Division of Hematology, Hematologic Malignancies, and Stem Cell Transplantation, University of Colorado, Denver, Colorado, United States of America; 5 Department of Pediatrics, Virginia Commonwealth University, Richmond, Virginia, United States of America,; Wayne State University School of Medicine, United States of America

## Abstract

Thiamine-dependent enzymes (TDEs) control metabolic pathways that are frequently altered in cancer and therefore present cancer-relevant targets. We have previously shown that the recombinant enzyme thiaminase cleaves and depletes intracellular thiamine, has growth inhibitory activity against leukemia and breast cancer cell lines, and that its growth inhibitory effects were reversed in leukemia cell lines by rapamycin. Now, we first show further evidence of thiaminase therapeutic potential by demonstrating its activity against breast and leukemia xenografts, and against a primary leukemia xenograft. We therefore further explored the metabolic effects of thiaminase in combination with rapamycin in leukemia and breast cell lines. Thiaminase decreased oxygen consumption rate and increased extracellular acidification rate, consistent with the inhibitory effect of acute thiamine depletion on the activity of the TDEs pyruvate dehydrogenase and 2-oxoglutarate dehydrogenase complexes; these effects were reversed by rapamycin. Metabolomic studies demonstrated intracellular thiamine depletion and the presence of the thiazole cleavage product in thiaminase-treated cells, providing validation of the experimental procedures. Accumulation of ribose and ribulose in both cell lines support the thiaminase-mediated suppression of the TDE transketolase. Interestingly, thiaminase suppression of another TDE, branched chain amino ketoacid dehydrogenase (BCKDH), showed very different patterns in the two cell lines: in RS4 leukemia cells it led to an increase in BCKDH substrates, and in MCF-7 breast cancer cells it led to a decrease in BCKDH products. Immunoblot analyses showed corresponding differences in expression of BCKDH pathway enzymes, and partial protection of thiaminase growth inhibition by gabapentin indicated that BCKDH inhibition may be a mechanism of thiaminase-mediated toxicity. Surprisingly, most of thiaminase-mediated metabolomic effects were also reversed by rapamycin. Thus, these studies demonstrate that acute intracellular thiamine depletion by recombinant thiaminase results in metabolic changes in thiamine-dependent metabolism, and demonstrate a previously unrecognized role of mTOR signaling in the regulation of thiamine-dependent metabolism.

## Introduction

Thiamine (vitamin B1) is a cofactor for enzymes involved in critical metabolic processes involving energy production, biomass generation and amino acid catabolism. Despite the requirement for this vitamin in these central processes, the role of thiamine and thiamine-dependent enzymes (TDEs) in cancer development and treatment has received little attention, although a recent review has summarized the potential importance of TDE’s in cancer metabolism [1]. Unlike antifolates, which have a well-established role in cancer therapy, analogous small molecule thiamine antagonists are relatively inert, leading to a conclusion that TDE pathways could not be important as an anticancer targets. However, the limitations of small molecule TDE inhibitors should not be confused with the potential role of TDEs as anticancer therapeutic targets. Antifolates can be effective because intracellular folates only transiently associate with enzymes during the catalytic process, allowing for inhibition of enzyme activity by molecules designed to bind more tightly than the intracellular substrates. In contrast, intracellular thiamine, activated by phosphorylation, remains tightly bound to enzyme complexes during the catalytic cycle, leaving little opportunity for inhibitors to displace it once the complex has assembled. This inherent pharmacologic challenge could disguise the potential of targeting TDEs for cancer therapy.

We have previously shown down-regulation of thiamine transporter gene expression in tumors compared to normal tissues [2], [3] and more recently have shown that a low thiamine diet delays onset of mammary tumors in MMTV(her2) mice [4], an effect that is abrogated by a high fat diet. These observations have led to our hypothesis that TDE pathways are altered as part of the overall changes in energy metabolism that occurs in cancer cells, and that these changes could produce metabolic vulnerabilities that could be exploited by therapies aimed at TDE activities. To take a novel path in the exploration of TDEs in cancer, we have studied the cytotoxic activity of the bacterial enzyme thiaminase, which cleaves thiamine into its pyrimidine and thiazole moieties [5]. Thiaminase overcomes the limitations of small molecule TDE inhibitors by causing immediate and nearly complete intracellular and extracellular thiamine deprivation [6]. In previous studies we have shown that thiaminase has both *in vitro* and *in vivo* cytotoxicity activity, further supporting the concept that TDEs could represent new targets for novel therapies [6], [7], [8]. We have also previously reported that rapamycin has antagonistic effect on thiaminase-mediated growth inhibition of leukemia cells [7], a surprising finding since rapamycin generally acts as a sensitizing agent in combination with cytotoxic drugs.

We now present metabolic and metabolomic observations regarding the anticancer activities and metabolic effects of thiaminase in leukemia and breast cancer cells. We chose to focus on breast and leukemia models because these were the models in which we observed the most promising activity of thiaminase in xenografts. These studies help define thiaminase metabolic effects that may be responsible for its cytotoxic activity. These studies also further elucidate the role of mTOR as an inhibitor of thiaminase-mediated alterations in cellular metabolism, and demonstrate the role of mTOR in regulating expression of enzymes involved in thiamine-dependent metabolism.

## Methods

### Ethics statement

All animal studies were approved by the University of Kentucky Institutional Animal Care and Use Committee.

### Cell Lines

The human breast cancer cell line MCF-7 and the non-malignant breast cell line MCF-10A were obtained from ATCC; human leukemia cell lines Reh and RS4 were originally obtained from ATCC and generously provided by Dr. Terzah Horton, Baylor College of Medicine. Cell line authentication was performed by PCR amplification of nine short tandem repeat (STR) loci (Research Animal Diagnostic Laboratory, St. Louis, MO) and comparing the profile to the ATCC STR database. The STR profile of MCF-7 and RS4 cell lines were identical to the ATCC profile. The Reh cell line matched all alleles in the ATCC Reh profile plus one extra allele at two loci.

### Cytotoxicity assays

Human leukemia cell lines Reh or RS4 were plated in triplicate in 96-well microtiter plates in RPMI-1640 (with 25 mM HEPES) medium containing 10% fetal bovine serum at final densities of 8×10^4^ cells/well. MCF-7 cells were plated in the same medium at the final density of 1000cells/well. Medium containing native thiaminase at a concentration range of 1×10^−6^ to 4 units/ml was added to cells and incubated for four days. Following incubation, an MTT Cell Proliferation Assay (ATCC) was performed according to the ATCC protocol. The IC_50_ was calculated from the dose response curve as the concentration of drug producing a 50% decrease in the mean absorbance compared to the untreated wells using Prism GraphPad software. The cytotoxicity experiments were repeated a minimum of three times in triplicate. Analysis of primary human ALL specimens was performed by plating cells at a density of 1×10^6^/ml and treating for 24 hours with the indicated concentration of thiaminase. Viability was evaluated by dead cell exclusion labeling with trypan blue dye, as well as flow cytometric analyses using Annexin-V labeling as previously described [9].

### Xenograft studies

RS4 tumor xenografts were established by subcutaneously inoculating 1×10^7^ cells into the right flank of five-week-old female Crl:NU-Foxn1 nude mice (Charles River Laboratories, Wilmington, MA). For the establishment of MCF-7 xenograft, a 17β-estradiol pellet (0.72 mg, 60 days release; Innovative Research of America, Sarasota, FL) was implanted subcutaneously into the neck to facilitate optimal tumor growth for the estrogen receptor–positive MCF-7 cells. The xenografts were initiated by subcutaneously injecting 5×10^6^ MCF-7 cells into the right flank of five-week-old female Crl:NU-Foxn1 nude mice. When palpable tumors had formed, mice were treated with native thiaminase at its MTD (2000 units/kg body weight) twice a week at a site distant from the formed tumor for three weeks. Mice were treated with a single dose of thiaminase enzyme conjugated with 1k linear chain PEG (1K-LCPTE) at its MTD (50 U/kg) at a site distant from the formed tumor. Mice treated with N3-pyridyl thiamine (N3PT) received an intraperitoneal (i.p.) dosage of 80 mg/kg daily for five days. Mice treated with both 1K-LCPTE and N3PT received a single dose of 1K-LCPTE at 50 U/kg first, then after 5five days, mice received N3PT at 80 mg/kg daily for five days. Tumors were measured twice a week in a blinded manner by measuring perpendicular diameters with a digital caliper and tumor volumes (mm^3^) were calculated using the following formula: volume  =  width × width × length × π/6. The predetermined endpoint was a tumor volume of 1500 mm^3^. The control mice were injected with MCF-7 or RS4 cells and left untreated, and results combined with a previous control group of the same xenograft [7]. Kaplan-Meier survival curves and statistical analysis was performed with GraphPad Prism software.

### Primary lymphoblastic leukemia xenograft methods

A primary lymphoblastic leukemia specimen was transplanted by IV injection into sub-lethally irradiated immune deficient NOD/SCID/IL2Rg mice. When the tumor burden was established in the bone marrow (day 17), animals received treatment with thiaminase 2000 units/kg on days 17, 20 and 24, administered by subcutaneous injection (the longer interval between treatments in these mice in comparison to the Crl:NU-Foxn1 nude mice was due to tolerability). The animals were sacrificed on day 33 and marrow cells were isolated and examined by flow cytometry using human-specific antibodies for CD45 and CD19 to determine the level of human leukemia cell engraftment as previously described [9].

### Mitochondrial bioenergetics measurements

Oxygen consumption was determined using the Seahorse Extracellular Flux (XF-96) analyzer (Seahorse Bioscience, Chicopee, MA). The XF-96 measures the concentration of oxygen and free protons in the medium above a monolayer of cells in real-time. Thus, the rates of oxygen consumption and proton production can be measured across several samples at a time. To allow comparison between experiments, data are presented as oxygen consumption rate (OCR) in pMoles/min/10^4^ cells and the extracellular acidification rate (ECAR) in mpH/min/10^4^ cells. RS4 and Reh leukemia cells were seeded at 125,000 cells/well into gelatin-coated Seahorse Bioscience XF microplates, cultured in the presence or absence of 2 g/L D-glucose, and then centrifuged to adhere to the bottom of the wells, while for the MCF-7 and MCF-10A about 45,000 cells were plated and allowed to adhere overnight. OCR was measured four times and plotted as a function of cells under the basal condition followed by the sequential addition of oligomycin (1 µg/ml), FCCP (1 µM) and rotonone (1 µM). The ATP-linked OCR was calculated as the basal OCR minus the OCR measured after the addition of oligomycin. The OCR maximal capacity was the direct rate measured after the addition of FCCP. The reserve capacity is the FCCP OCR minus the basal OCR. For the ECAR measurements, cells were washed following overnight incubation and changed to assay media lacking glucose. Basal ECAR were measured four times and plotted as a function of cells under the basal condition followed by the sequential addition of glucose (25 mM), oligomycin (1 µg/ml) and 2-deoxyglucose (25 mM). The rate of glycolysis was determined by subtracting the basal ECAR from the ECAR after the addition of glucose. Glycolytic reserve was determined by subtracting the ECAR following the addition of oligomycin from the ECAR following the addition of glucose. Differences between treatment groups were calculated using the Kruskal Wallis test.

### Metabolomic studies

RS4 leukemia cells and MCF-7 breast cancer cells were analyzed under six conditions: control for 24 hrs (C-24); incubation in thiaminase for 24 hrs (T-24); control for 48 hours (C-48); thiaminase for 48 hrs (T-48); rapamycin for 48 hrs (R-48); and both rapamycin and thiaminase for 48 hrs (R+T-48). Four independent samples were produced for each time point for each cell line, and cell pellets were stored at −80°C until the separate experiments were all completed. Metabolomic studies were performed at Metabolon, Inc. (Durham, NC). The non-targeted metabolic profiling platform consisted of three independent instrumental methods: ultrahigh performance liquid chromatography/tandem mass spectrometry (UHPLC/MS/MS^2^) optimized for basic species; UHPLC/MS/MS^2^ optimized for acidic species; and gas chromatography/mass spectrometry (GC/MS). The detailed process of the platform; including sample processing, instrument configuration, data acquisition, as well as metabolite identification and quantitation, were published previously [10], [11]. Three hundred and forty two metabolites were identified by automated comparison of the ion features in the experimental samples to a reference library of chemical standard entries that included retention time, molecular weight (*m/z*), preferred adducts, in-source fragments and associated MS spectra [12]. Instrument variability was determined by calculating the median relative standard deviation (RSD) for the internal standards that were added to each sample prior to injection into the mass spectrometers. Overall process variability was determined by calculating the median RSD for all endogenous metabolites (i.e., non-instrument standards) present in 100% of a set of technical replicates of pooled samples. Values for instrument and process variability meet Metabolon’s acceptance criteria with instrument variability of 3% and overall process variability of 12%. Following normalization to total protein (Bradford assay), log transformation and imputation with minimum observed values for each compound, Welch’s two-sample *t*-tests were used to identify biochemicals that differed significantly between experimental groups. The entire metabolomic data sets for RS4 and MCF-7 cells, with statistical results, are included in data tables (Table S1).

### Immunoblot analysis

Cells were treated with thiaminase (0.001 U/ml) and/or rapamycin (0.1 µM) for 96 hours. Cells were lysed with a triple-detergent lysis buffer (50 mM Tris pH8.0, 150 mM NaCl, 1% NP-40, 0.5% DOC, 0.1% SDS, 0.02% sodium azide, 100 µg/ml PMSF, protease inhibitors (Roche) and phosphatase inhibitors (Thermo Scientific)). Equal amounts of protein were loaded into each well and separated by SDS-PAGE gel, followed by transfer onto nitrocellulose membranes. The membranes were blocked, incubated with the indicated primary antibodies at 4°C overnight, and the appropriate horseradish peroxidase–conjugated secondary antibody was added for 1 hour at room temperature. Immunoblots were developed by use of SuperSignal West Pico chemiluminescent substrate (Thermo Scientific) according to the manufacturer’s protocol and analyzed by FujiFilm LAS-4000 luminescent image analyzer (Multigauge software). The primary and secondary antibodies used in this study are listed as follows. Anti- PKM1; anti-BCAT2; anti-TPK1 and anti-THTPA antibodies were purchased from Proteintech Group, Inc. (Chicago, IL). Anti- PKM2 and anti-CPT1A antibodies were from Cell Signaling Technology (Danvers, MA). Anti-BCKDE1 and anti-phospho-BCKDE1 antibodies were obtained from Bethyl laboratories (Montgomery, TX). Anti-BCAT1, anti-β-actin and all secondary antibodies were obtained from Sigma-Aldrich (St. Louis, MO).

## Results

**Figure 1:**
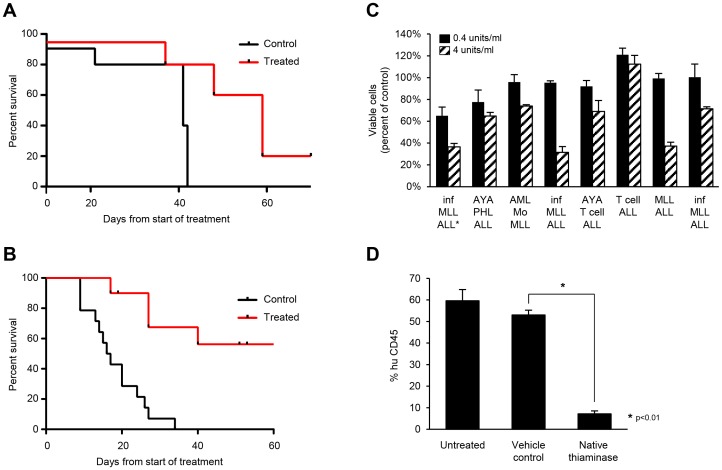
In vivo evidence of thiaminase anticancer activity. A. A Kaplan-Meyer plot of time to pre-defined tumor volume endpoint for subcutaneous MCF-7 breast cancer xenografts treated with thiaminase 2000 units SC QOD or buffer control. The median time to endpoint was 41 days for untreated control and 59 days (p = 0.03 Log rank test). B. A Kaplan-Meyer plot of time to pre-defined tumor volume endpoint for subcutaneous RS4 leukemia subcutaneous xenografts treated with thiaminase 850 units SC BIW or buffer control. The median time to endpoint was 16.5 days for the control group and not reached after 60 days of observation in the treated group (p<0.001 Log rank test). C. Primary ALL and AML specimens were thawed and plated in triplicate in two concentrations of thiaminase 0.4 units/ml and 4 units/ml, and assessed for viability at 48 hours relative to untreated cells. The ALL specimen with the asterisk was used for the *in vivo* study shown in Figure 1D. D. Primary ALL cells were injected IV on Day 1; three thiaminase treatments of 2000 units/kg SC were administered on days 17, 20 and 24. The animals were sacrificed on Day 33 and bone marrow was examined by flow cytometry for human ALL cells (percent human CD45); Untreated n = 4; vehicle treated n = 10, native thiaminase n = 8 (* p<0.01, Mann-Whitney test).

Evidence of antitumor activity of thiaminase in leukemia and breast cancer tumor models is shown in Figure 1. Figure 1A is a Kaplan-Meier plot of MCF-7 subcutaneous xenografts treated with thiaminase, showing a prolongation in the time to endpoint (pre-defined tumor volume) (TTE) from 41 days in the mock treated cohort to 59 days in the treated cohort (p = 0.03). In Figure 1B RS4 subcutaneous xenografts show an increase in median TTE from 16.5 days from the start of treatment to undefined TTE after 60 days (p<0.001). We have also previously shown evidence of thiaminase activity against MDA231 breast cancer [8]. In Figure 1C, the activity of thiaminase is shown against primary human leukemia cells. The most sensitive primary leukemia specimens appear to be lymphoblastic specimens with MLL-gene re-arrangements. This was of interest as the RS4 cell line is also an MLL-rearranged cell line. Figure 1D shows flow cytometric analysis of bone marrow of a primary MLL-rearranged leukemia cell xenograft treated with thiaminase demonstrating a decrease in leukemia cell proportion after treatment. These studies, along with previous studies [7], [8], provided the rationale for performing further detailed examination of the metabolic effects of thiaminase in the breast cancer cell line MCF-7 and in the RS4 leukemia cell line. In addition, for further points of comparison, we included selected studies in two additional cell lines, Reh leukemia cells, another lymphoblastic leukemia cell line, and MCF-10A, a non-malignant breast cell line.

**Figure 2:**
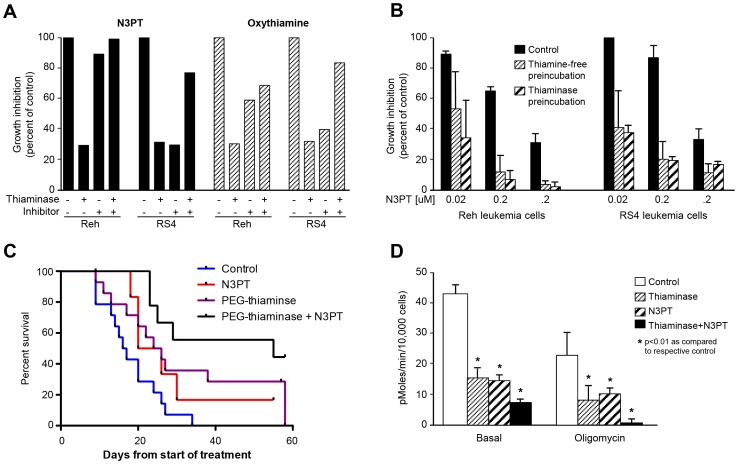
Effect of thiamine antagonists on thiaminase activity. A. Growth inhibition of Reh and RS4 leukemia cells in the presence or absence of thiaminase and either N3PT or oxythiamine. B. Growth inhibition of RS4 and Reh leukemia cells incubated in normal medium, thiamine-free medium or medium containing thiaminase prior to exposure to different concentrations of N3PT. C. RS4 subcutaneous xenografts showing untreated control, N3PT alone, 1k-PEGylated thiaminase and 1k-PEGylated thiaminase followed by N3PT. The time-to-endpoint was 16.5 days for control, 23 days for N3PT, 25 days for 1k-PEG thiaminase and 55 days for 1k-PEG thiaminase (p<0.01 Log rank test). D. Oxygen consumption rate of RS4 cells treated with thiaminase, N3PT or thiaminase followed by N3PT (clear: control; thin-stripe: thiaminase; thick- stripe: N3PT; solid: thiaminase + N3PT).

The studies in Figure 1, along with previous reports, demonstrate that the enzyme thiaminase has activity against breast and lymphoid leukemia cell lines. To first determine whether the activity of thiaminase was related to thiamine depletion, we explored the ability of small molecule thiamine antagonists to potentiate the cytotoxicity of thiaminase. We screened thiamine analogs pyrithiamine, oxythiamine and N3’ pyridylthiamine (N3PT) [13], [14] for activity as inhibitors of thiaminase and substrates of thiaminase. We found that oxythiamine and N3PT were thiaminase inhibitors, and that pyrithiamine was a thiaminase substrate (data not shown). Therefore we hypothesized that oxythiamine and N3PT would reverse thiaminase toxicity if given simultaneous with thiaminase (because they would inhibit enzyme activity) but would act synergistically if given in sequence (because cells deprived of thiamine would become sensitive to small molecule thiamine antagonists due to TDE apoenzyme formation during thiamine starvation). In Figure 2A, simultaneous incubation of thiaminase and thiamine antagonists inhibits thiaminase activity, while in Figure 2B, sequential administration of thiaminase followed by N3PT demonstrates synergistic cytotoxicity. Importantly, the effect of pre-incubation in thiaminase is similar to the effect of pre-incubation in thiamine-free medium, indicating that TDE apoenzyme formation is required for the cytotoxicity of small molecule TDE inhibitors, and also demonstrating that extracellular thiamine starvation is sufficient to make small molecule TDE inhibitors cytotoxic. In order to further examine the interplay of N3PT and thiaminase in an *in vivo* model, we administered PEGylated thiaminase in sequence with N3PT, choosing PEGylated enzyme over the native enzyme shown in Figure 1B to be able to separate extracellular thiamine deprivation caused by PEGylated thiaminase from the intracellular effect of N3PT [6]. In Figure 2C, the RS4 xenograft experiment shows that sequential administration of PEGylated thiaminase followed by N3PT produced an increased median TTE (55 days) compared to the untreated control (16.5 days), PEG-thiaminase (25 days) or N3PT (23 days) alone (p<0.01), confirming the in vitro data.

**Figure 3:**
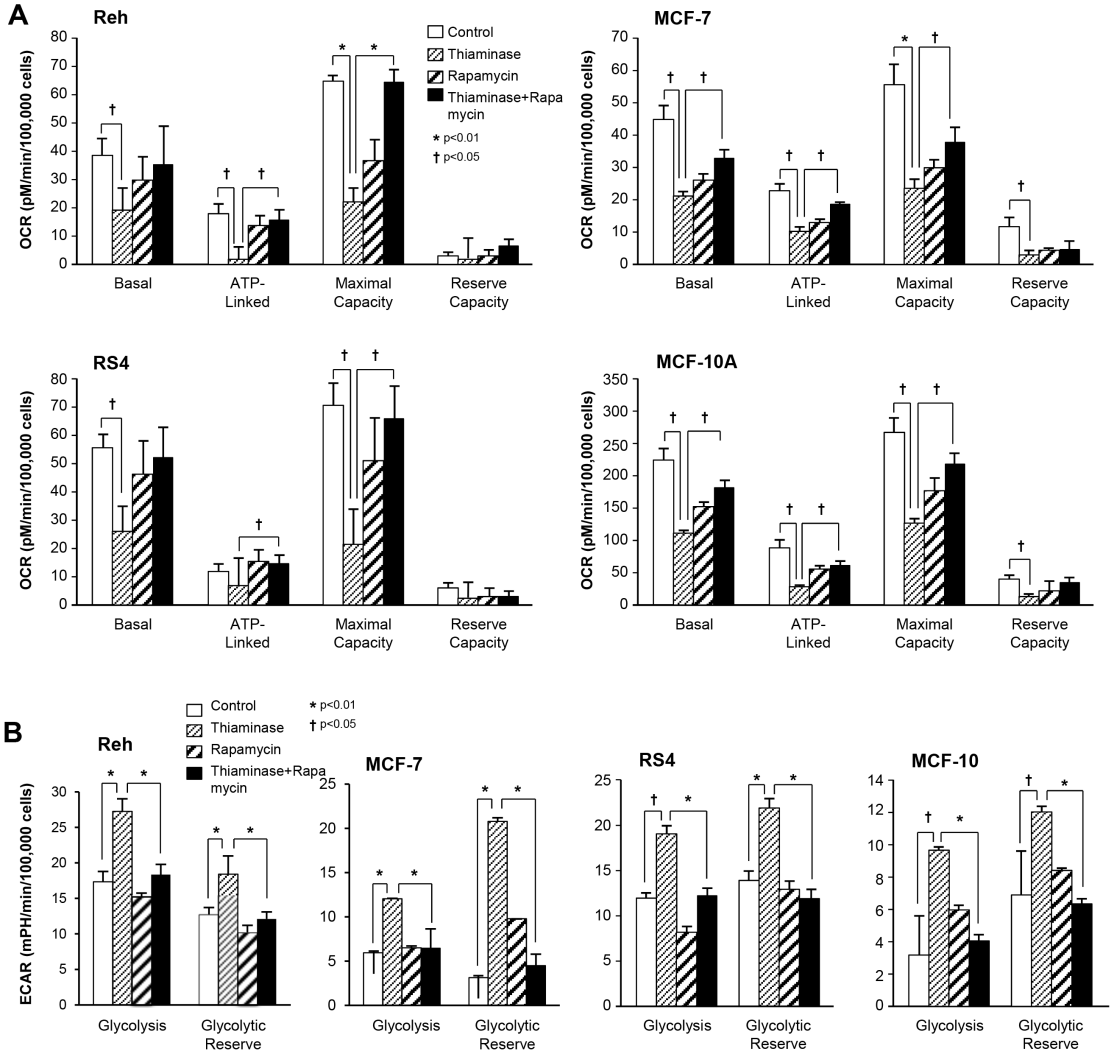
OCR and ECAR of leukemia and breast cell lines incubated for 48(clear: control; thin-stripe: thiaminase; thick- stripe: rapamycin; solid: thiaminase + rapamycin). A. Quantification of OCR parameters. The ATP-linked rate is the basal rate minus the rate measured after the addition of oligomycin. The maximal capacity is the rate measured after the addition of FCCP. The reserve capacity is the basal rate minus the FCCP rate. All data are the mean ± SEM, of triplicate samples and are representative of 3 independent experiments (†p<0.05, *p<0.01 two way ANOVA (Newman Kruskal Wallis test). B. Quantification of ECAR parameters. Glycolysis is the rate determine from subtracting the basal rate from the rate after the addition of glucose. Glycolytic reserve is the rate determined by subtracting the rate following the addition of oligomycin from the rate following the addition of glucose. All data are the mean ± SEM, of triplicate samples and are representative of 3 (†p<0.05, *p<0.01 two way ANOVA (Newman Kruskal Wallis test).

Since thiaminase should inhibit two key TDEs involved in Krebs cycle metabolism, pyruvate dehydrogenase complex and 2-oxoglutarate dehydrogenase (2-OGDH, aka alpha-ketoglutarate dehydrogenase), we examined respiration (as oxygen consumption rate; OCR) and the rate of extracellular acidification (a measure of lactate production; ECAR) in leukemia and breast cancer cell lines. In Figure 2D, the oxygen consumption rate is decreased in an additive fashion with the sequential administration of thiaminase followed by N3PT, showing further evidence of interaction of thiaminase and a small molecule thiamine antagonist. In addition, since rapamycin reversed thiaminase-mediated growth inhibition in leukemia cell lines [7] but not breast cancer cell lines (data not shown), we examined the effect of rapamycin alone and in combination with thiaminase. In Figure 3A, thiaminase consistently decreased OCR in two lymphoid leukemia cell lines RS4 and Reh, in the breast cancer cell line MCF-7 and in the non-malignant breast cell line MCF-10A. Also, as expected, rapamycin similarly suppressed OCR in the four cell lines. However, adding rapamycin resulted in an inhibition of the thiaminase-mediated decrease in OCR, rather than resulting in an additive effect as was seen with N3PT in Figure 2B.

Similarly, thiaminase increased ECAR in the two breast cell lines: the MCF-7 cell line and the MCF-10A cell line (Figure 3B). Although rapamycin alone did not have a significant effect on ECAR, the addition of rapamycin to thiaminase once again blunted the effect of thiaminase in all four cell lines. The consistent inhibition of thiaminase effect on OCR and ECAR by rapamycin in all four cell lines demonstrates a previously unidentified activity of mTOR inhibition in relation to TDEs. However, the observation that the rapamycin has similar effects on the leukemia cell lines, where it reverses growth inhibition [7], and MCF-7 and MCF-10A cell lines, where it has no effect on thiaminase cytotoxicity (data not shown), suggests that the effect of thiaminase on OCR and ECAR may not be the determinant of thiaminase anticancer activity. To rule out the possibility that rapamycin could directly inhibit thiaminase enzymatic activity, which could explain the reversal of the effects of thiaminase by rapamycin, we directly examined the ability of rapamycin to inhibit thiaminase enzyme activity and found no evidence that rapamycin was a thiaminase enzyme inhibitor (data not shown).

**Figure 4:**
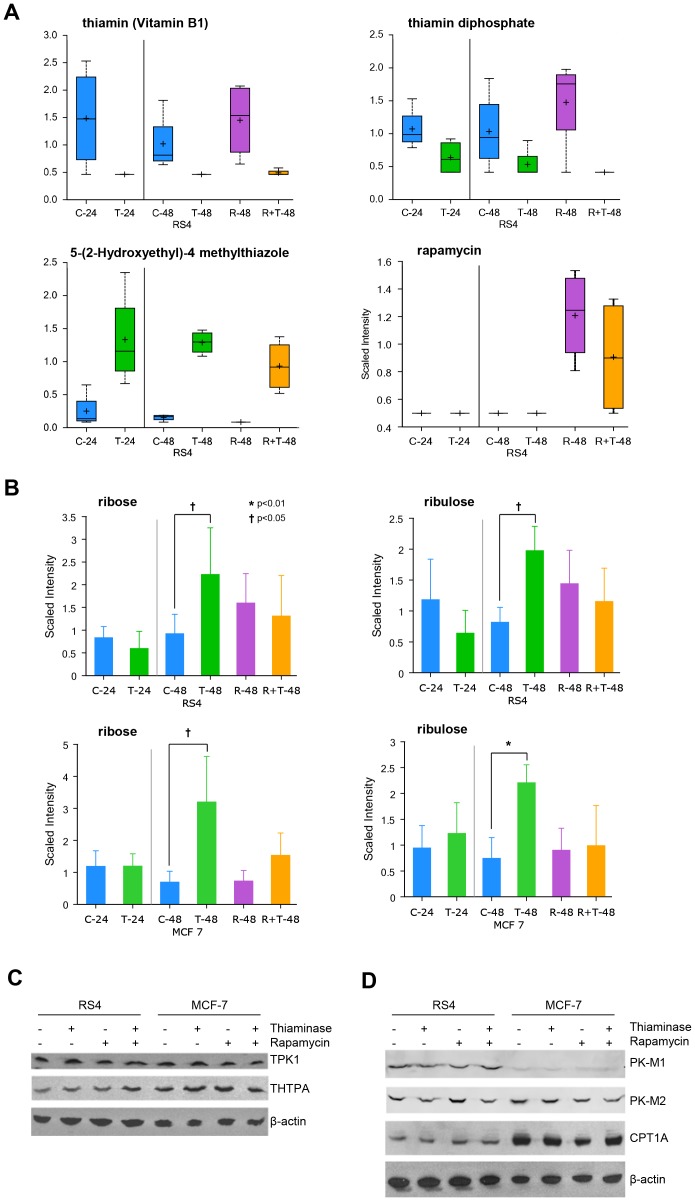
Validation of thiaminase action in of RS4 leukemia cells and MCF-7 breast cancer cells by metabolomic analysis. Both cell lines were analyzed under six conditions: control for 24 hours (C-24); incubation in thiaminase for 24 hours (T-24); control for 48 hours (C-48); thiaminase for 48 hours (T-48); rapamycin for 48 hours (R-48); and both rapamycin and thiaminase for 48 hours (R+T-48). The median is indicated by the bar in the center of the rectangle, the rectangle dimensions reflect the range of the two mid-quartile values, and the outer bars represent the ranges of all of the values. The data represent four independent experiments. For C-48 vs T-48 and C-48 vs T+R-48 comparisons, ** indicates p<0.05 and * indicates 0.05<p<0.1). A. Internal validation in RS4 cells, showing the expected decrease in thiamine and thiamine diphosphate in thiaminase treated cells, an increase in thiazole, the product of thiaminase cleavage of thiamine in the thiaminase-treated cells and the appearance of rapamycin only in the rapamycin treated cells. B. Validation of thiaminase-mediated inhibition of transketolase by demonstration of the accumulation of substrate surrogates ribose and ribulose in both RS4 and MCF-7 cell lines at 48 hours. C. Immunoblot analysis of thiamine pyrophosphate kinase (TPK1) and thiamine triphosphatase (THTPA) in RS4 and MCF-7 cells treated with thiaminase, rapamycin or both. D. Immunoblot analysis of pyruvate kinase isozymes M1 and M2 and carnitine palmitoyl transferase (CPT1) in RS4 and MCF-7 cells treated with thiaminase, rapamycin or both.

To further understand how thiaminase alters cellular metabolism, and how rapamycin reverses thiaminase effects, we undertook a metabolomic analysis of RS4 leukemia cells and MCF-7 breast cancer cells exposed to thiaminase, rapamycin or both. Figure 4A shows internal validation of the effects of thiaminase in RS4 cells. As expected, the levels of thiamine and thiamine diphosphate were decreased in the thiaminase treated conditions, and thiazole (a catabolic product of thiaminase cleavage of thiamine) is increased in the thiaminase treated cells, confirming the finding that rapamycin does not interfere with the enzymatic activity of thiaminase. In fact, thiamine was below the limit of detection in thiaminase treated cells in the absence of rapamycin. Also, rapamycin was detected only in the rapamycin-treated cells. Evidence for inhibition of the TDE transketolase was observed with significant increases in ribose and ribulose, surrogates for transketolase substrates ribose 5-phosphate and ribulose 5-phosphate respectively, in both RS4 and MCF-7 cells (Figure 4B). These results demonstrate a common signature of transketolase inhibition in the two cell lines. Furthermore, the accumulation is partly to completely reversed by rapamycin, demonstrating an antagonistic effect of rapamycin on thiamine-induced transketolase inhibition in addition to its antagonistic effects on OCR and ECAR.

Although there was no evidence that rapamycin altered the level of thiaminase-induced thiamine depletion, we sought to confirm this observation by examining the expression of enzymes that regulate thiamine phosphorylation. In Figure 4C the expression of both thiamine pyrophosphokinase TPK1, an enzyme that phosphorylates thiamine and thiamine monophosphate into its active diphosphate form, and thiamine triphosphatase (THTPA), an enzyme that dephosphorylates intracellular thiamine triphosphate into the active diphosphate form, are shown. A modest increase in THTPA expression is seen in RS4 cells treated with both thiaminase and rapamycin, which may be a result of feedback from intracellular thiamine diphosphate depletion, but no other changes are seen in either RS4 or MCF-7 cells. In Figure 4D, the expression of enzymes involved in energy metabolism, pyruvate kinase isoforms M1 and M2, and carnitine palmitoyl transferase 1 (CPT1A) were also examined by immunoblot. PK-M1 expression was decreased in MCF-7 cells relative to RS4 cells. In MCF-7 cells, both PK-M2 and CPT1A expression levels were modestly reduced by rapamycin, and this suppression was reversed by thiaminase in the case of CPT1A but not PK-M2. However, similar findings were not observed in RS4 cell lines, adding more evidence to the overall observation that different cell lines will respond differently to acute thiamine depletion.

**Figure 5:**
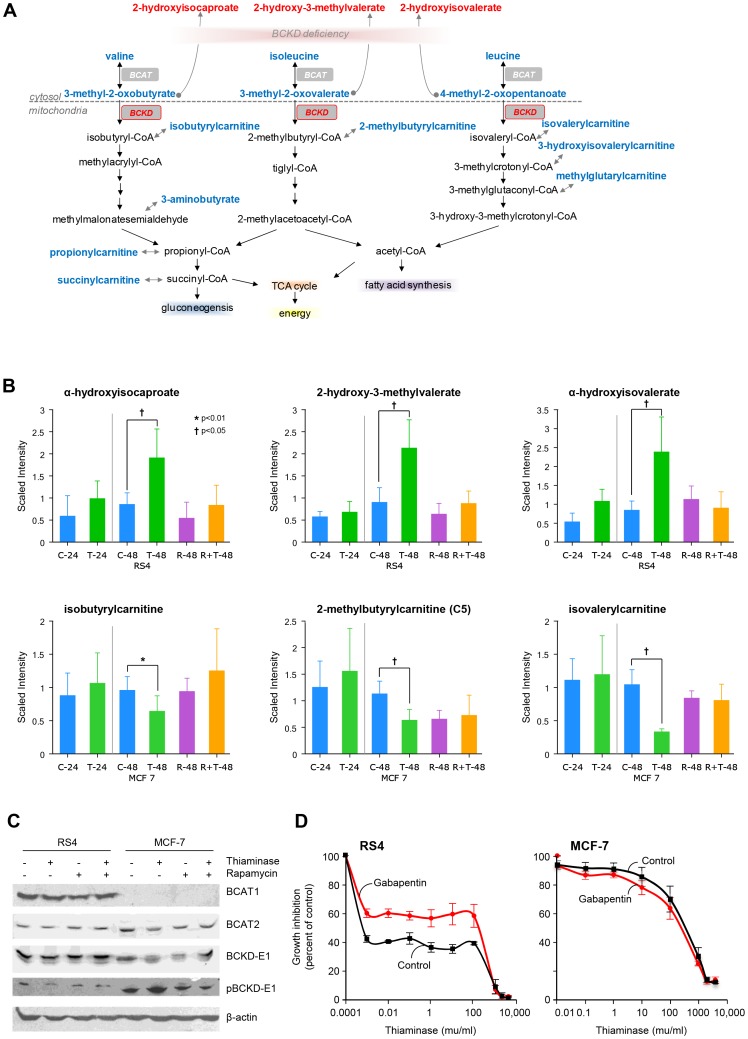
Differential effects of thiaminase on branched chain amino acid catabolism. A. Schematic diagram showing branched chain amino acid catabolism. B. Different metabolomic signatures indicating inhibition of BCKDH by thiaminase. The top three panels show accumulation of BCKDH substrates after 48-7 cells after thiaminase treatment- most notably isovalerylcarnitine, which is also reversed by rapamycin. C. An immunoblot of cytosolic and mitochondrial branched chain amino acid transferase (cBCAT and mBCAT), the enzymes that catalyzes the reactions that produce BCKDH substrates, and total and phosphorylated BCKDH subunit E1 (BCKD-E1 and pBCKD-E1, respectively) in RS4 leukemia cells treated with thiaminase, rapamycin or both. For C-48 vs T-48 and C-48 vs T+R-48 comparisons, ** indicates p<0.05 and * indicates 0.05<p<0.1). D. Cytotoxicity assay of RS4 and MCF-7 cells treated with increasing concentrations of thiaminase (in milliunits, mu) under control conditions or in medium that contains gabapentin.

Figure 5 shows effects of thiaminase on the TDE branched-chain keto acid dehydrogenase (BCKDH), and demonstrates different metabolomic signatures between RS4 leukemia cells and MCF-7 breast cancer cells. In Figure 5A the metabolic pathways for the catabolism of branched chain amino acids is illustrated. The metabolomic profile for these metabolites is shown in Figure 5B and Figure S1. In RS4 cells, thiaminase increases the levels of BCKDH substrates alpha-hydroxyisocaproate, 2-hydroxy-3-methylvalerate and alpha-hydroxyisovalerate, all metabolites that are increased in maple syrup urine disease, the congenital form of BCKDH deficiency. In contrast, a decrease in the products of BCKDH are observed in MCF-7 cells, with decreased isobutyrylcarnitine, 2-methybutyrylcarnintine and isovalerylcarnitine. Once again the effects of thiaminase were offset by rapamycin. These findings suggest that the substrate load for BCKDH is much higher in RS4 leukemia cells. Therefore, we investigated the expression of the cytosolic form and the mitochondrial form of the branched chain aminotransferases, cBCAT and mBCAT - the enzymes immediately upstream of BCKDH, in both RS4 and MCF-7 cells. As shown in Figure 5C, MCF-7 cells do not appear to express cBCAT, providing a potential explanation for the lack of BCKDH substrate accumulation in this cell line after thiaminase treatment. Figure 5C also appears to show an apparent increase in phosphorylated BCKD-E1 after thiaminase treatment in MCF-7 cells which is reversed by co-treatment with rapamycin. Phosphorylation of BCKDH inactivates the enzyme and may contribute to the decrease of BCKDH products seen in the MCF-7 cells, and suggests that there may be an interaction between thiaminase, rapamycin and the enzymes that regulate BCKDH phosphorylation. To determine whether the accumulation of BCKDH substrates contributed to thiaminase-induced growth inhibition in RS4 cells, we examined thiaminase growth inhibition in the presence of gabapentin, a structural analog of leucine and an inhibitor of cBCAT [15]. As shown in Figure 5D, gabapentin partially protected RS4 cells from thiaminase growth inhibition while having no effect on MCF-7 cells.

**Figure 6:**
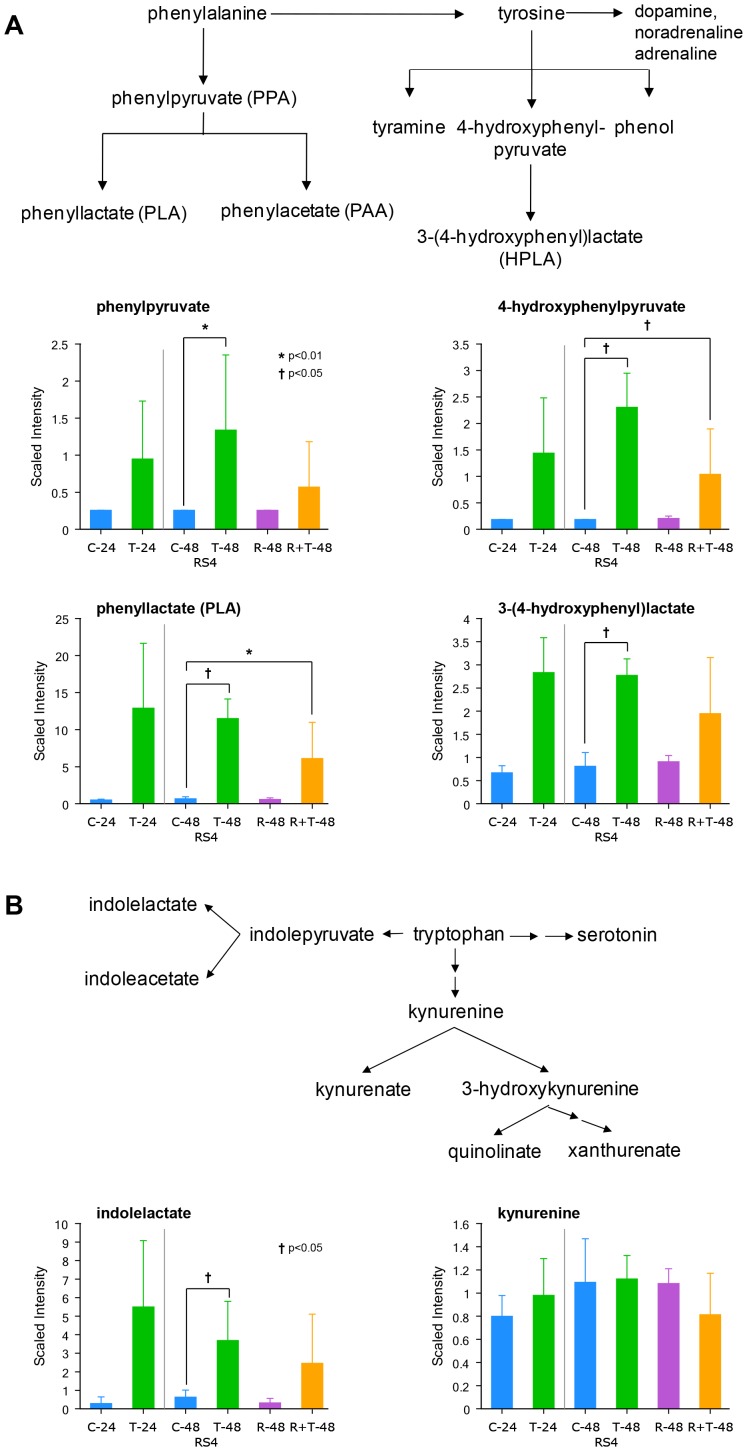
Effects of thiaminase on aromatic amino acid catabolism. A. Accumulation of the products of phenylalanine (phenylpyruvate and phenyllactate) and tyrosine (hydroxyphenylpyruvate and hydroxyphenyllactate) catabolism in RS4 cells after treatment with thiaminase for 48 hours. B. Accumulation of tryptophan catabolites after thiaminase treatment, showing accumulation of indolelactate but not kynurenine. For C-48 vs T-48 and C-48 vs T+R-48 comparisons, ** indicates p<0.05 and * indicates 0.05<p<0.1).

Thiaminase also affected the catabolism of aromatic amino acids in RS4 leukemia cells. As shown in Figure 6A, the breakdown products of both phenylalanine (phenylpyruvate and phenyllactate) and tyrosine (4-hydroxyphenylpyruvate (HPP) and 3-(4-hydroxyphenyl)lactate (HPLA)) are increased in the thiaminase treated cells. Furthermore, as shown in Figure 6B, tryptophan catabolites are increased through one pathway which produces indolelactate, but not through the kynurenine pathway. As in the approach we took with the alteration of branched chain amino acids, we examined the possibility that the accumulation of these aromatic amino acid catabolites mediated the growth inhibitory effects of thiaminase. The drug nitisinone, which inhibits 4-hydroxyphenylpyruvate dioxygenase, an enzyme that catalyzes an alternative catabolic pathway for phenylalanine [16] and would be expected to increase the accumulation of aromatic amino acid catabolities, did not however alter the dose-response curves of thiaminase in either cell line (data not shown).

## Discussion

Any systematic difference between cancer cells and normal cells provides a potentially exploitable therapeutic opportunity. The altered energy metabolism in cancer cells, known as the Warburg effect, represents such an opportunity. The vitamin thiamine is a key cofactor in two critical enzymes in energy metabolism: PDH and 2-OGDH. It is also a cofactor for two other critical enzymes; TK, which is required for the formation of the biomass of a cell through the pentose phosphate shunt; and BCKDH which regulates the catalysis of branched chain amino acids.

Thiamine requires active transport for cellular uptake. We have previously shown down-regulation of thiamine transporters in human tumors [2], [3], suggesting that tumors may have a nutritional vulnerability that could be exploited clinically, analogous to the down-regulation of asparagine synthase in acute lymphoblastic leukemia which is exploited therapeutically by the bacterial enzyme asparaginase. The enzyme thiaminase catalyzes the cleavage of thiamine into two molecules: one is a pyrimidine (conjugated to a nucleophile for type I thiaminases) and the other is a thiazole that may be phosphorylated, depending on the thiamine substrate that enters the reaction [5]. Several forms of thiaminase exist in nature, including plant, animal and bacterial forms of the enzyme, thus serving as an example of convergent evolution even though the physiologic role of an enzyme that destroys an essential nutrient is not clear. We have developed methods to produce, purify and modify *Bacillus thiaminolyticus* thiaminase I enzyme and have shown in preclinical xenograft models that therapy directed at thiamine dependent enzymes (TDEs), a completely novel concept and strategy, has the potential to treat leukemia [7]and breast cancer [8], and despite its anti-metabolite role, thiaminase can cause tumor responses at a systemically tolerable dose.

To determine which TDE is responsible for the cytotoxic effects of thiaminase we examined the effect of the enzyme on cellular respiration and metabolism with the goal of identifying the critical TDEs responsible for cytotoxicity in the setting of acute thiamine deprivation. Since thiaminase is a bacterial enzyme, it is less-than-ideal as a pharmaceutical product and the identification of the specific pathway disrupted by thiamine deprivation that leads to cytotoxicity could reveal an alternative pharmacologic approach. The metabolomic changes in the global biochemical profiles of RS4 leukemia cells and MCF-7 breast cancer cells treated with thiaminase are consistent with inhibition of BCKDH complex, pyruvate dehydrogenase complex, and transketolase inhibition. However, inhibition of the same enzyme was shown to result in different metabolic consequences in the case of BCKDH, where inhibition in RS4 cells caused a demonstrable increase in BCKDH substrates, whereas in MCF-7 cells inhibition led to a decrease in the products of the enzymatic reaction. This difference may then lead to different consequences on cell growth, as the substrates for BCKDH are known to be toxic metabolites and result in the organ toxicity in the metabolic syndrome maple syrup urine disease. We have previously shown that RS4 cells, as well as other lymphoid leukemia cell lines, have a biphasic dose response to thiaminase, where there is an initial growth inhibition related to extracellular thiamine depletion and a cytotoxic response at higher concentrations related to intracellular thiamine depletion [7]. Gabapentin appears to be protective against the growth inhibitory effects of thiaminase in RS4 cells at the concentrations that produce extracellular thiamine depletion, indicating that growth inhibition is not due solely to thiamine depletion but also to the accumulation of branched-chain amino acid catabolites. These studies demonstrate that the cell lines, and presumable tumors, have different metabolic consequences resulting from inhibition of the same enzymes, and that these consequences may form the basis of sensitivity to TDE inhibition as well as identify potential biomarkers of response.

In addition, thiaminase treatment was associated with increased lipolysis and decreased polyamine synthesis (data not shown), both of which were reversed by co-treatment with rapamycin. These changes are consistent with altered energy production and reduced cellular proliferation as a result of thiamine depletion, but also may be related to our previous observation that mammary tumor formation is delayed in mice fed a low-thiamine normal-fat diet but not in mice fed a low-thiamine high-fat diet [4]. Although fatty acid metabolism may be affected by inhibition of 2-hydroxyl-CoA lyase (HACL1), a peroxisomal enzyme that also requires thiamine pyrophosphate as a cofactor and mediates the catabolism of branched chain fatty acids and 2-hydroxyl straight chain fatty acids [17], we did not see any evidence for accumulation of these metabolites in either cell line.

## Conclusions

Overall these studies demonstrate that TDE disruption leads to specific metabolic consequences that are predictable from its role as a cofactor for enzymes that catalyze important steps in energy production, biomass generation and amino acid catabolism. Comparison of the changes observed with thiaminase treatment in RS4 cells versus MCF-7 cells reveals different biochemical signatures as a result of thiamine depletion that may reflect differences in the relative contributions of different pathways to energy production and anaplerotic contributions to the TCA cycle. The near-global reversal of the specific metabolic effects of thiaminase by rapamycin indicate a closer regulation of thiamine-dependent metabolism by mTOR than has been previously appreciated, and further studies are needed to determine how mTOR signaling pathways regulates thiamine-dependent metabolism. The disruption of thiamine metabolism results in cytotoxicity in breast cancer and leukemia tumor model systems, and has the potential to provide novel targets for therapies directed toward these malignancies.

## Supporting Information

Figure S1 **Results of metabolomic analysis for branched-chain amino acid metabolites in A.) RS4 cell line and B.)** MCF7 cell cline analyzed under six conditions: control for 24 hours (C-24); incubation in thiaminase for 24 hours (T-24); control for 48 hours (C-48); thiaminase for 48 hours (T-48); rapamycin for 48 hours (R-48); and both rapamycin and thiaminase for 48 hours (R+T-48). The median is indicated by the bar in the center of the rectangle, the rectangle dimensions reflect the range of the two mid-quartile values, and the outer bars represent the ranges of all of the values. The data represent four independent experiments. For C-48 vs T-48 and C-48 vs T+R-48 comparisons, ** indicates p<0.05 and * indicates 0.05<p<0.1).
(TIF)

Table S1 **Metabolomic pathway heat map data for RS4 and MCF-7 cells treated with thiaminase (T), rapamycin (R), or both (R+T) for 24 and 48 hours.**
(XLSX)
