# Supplementary figures and images for: Metabolic Effects of Acute Thiamine Depletion Are Reversed by Rapamycin in Breast and Leukemia Cells
Source: PLoS One. 2014 Jan 15;9(1):e85702. doi: 10.1371/journal.pone.0085702 (PMC3893258; doi:10.1371/journal.pone.0085702)

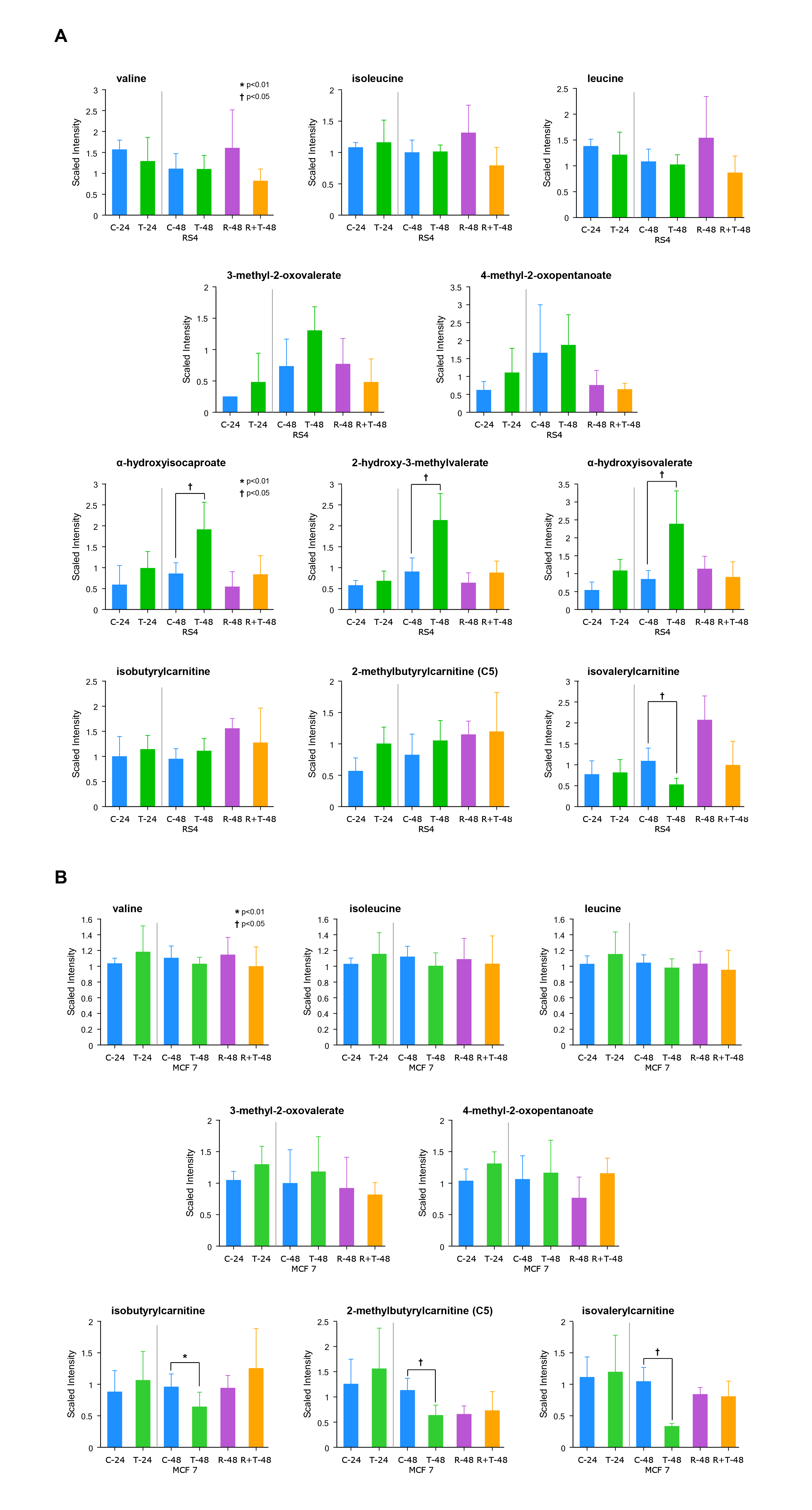

Supplement: Figure S1 — Results of metabolomic analysis for branched-chain amino acid metabolites in A.) RS4 cell line and B.) MCF7 cell cline analyzed under six conditions: control for 24 hours (C-24); incubation in thiaminase for 24 hours (T-24); control for 48 hours (C-48); thiaminase for 48 hours (T-48); rapamycin for 48 hours (R-48); and both rapamycin and thiaminase for 48 hours (R+T-48). The median is indicated by the bar in the center of the rectangle, the rectangle dimensions reflect the range of the two mid-quartile values, and the outer bars represent the ranges of all of the values. The data represent four independent experiments. For C-48 vs T-48 and C-48 vs T+R-48 comparisons, ** indicates p<0.05 and * indicates 0.05<p<0.1). (TIF) [file pone.0085702.s001.tif]
